# “Why shouldn’t I expect a lot from life?” – a qualitative study of what facilitates long-term recovery in first-episode psychosis

**DOI:** 10.1186/s12888-025-06681-y

**Published:** 2025-04-28

**Authors:** Gina Åsbø, Hanne Haavind, Sindre Hembre Kruse, Kristin Fjelnseth Wold, Wenche ten Velden Hegelstad, Kristin Lie Romm, Mike Slade, Torill Ueland, Ingrid Melle, Carmen Simonsen

**Affiliations:** 1 Section for Clinical Psychosis Research, Department of Research and Innovation, Division of Mental Health and Addiction, Oslo University Hospital, Oslo, Norway; 2 Department of Psychology, Faculty of Social Sciences, University of Oslo, Oslo, Norway; 3 Institute of Clinical Medicine, University of Oslo, Oslo, Norway; 4 Early Intervention in Psychosis Advisory Unit for Southeast Norway, Division of Mental Health and Addiction, Oslo University Hospital, Oslo, Norway; 5 TIPS – Centre for Clinical Research in Psychosis, Stavanger University Hospital, Stavanger, Norway; 6 Faculty of Social Sciences, University of Stavanger, Stavanger, Norway; 7 School of Health Sciences, Institute of Mental Health, University of Nottingham, Nottingham, UK; 8 Faculty of Nursing and Health Sciences, Health and Community Participation Division, Nord University, Namsos, Norway

**Keywords:** First-episode psychosis, Schizophrenia, Bipolar disorder, Recovery, Personal recovery, Qualitative

## Abstract

**Background:**

Qualitative research frequently *characterises* recovery, but more knowledge on subjective experiences of *facilitators* of *long-term* recovery in psychosis is needed. This interview study aimed to explore what people with first-episode psychosis (FEP) highlight as important for their long-term recovery.

**Methods:**

Interviews with 20 individuals in recovery (personal and/or clinical) participating in two follow-up studies, 10 and 20-years after treatment start for a first episode schizophrenia or bipolar spectrum disorder. Interviews were thematically analysed by a research team that included a peer researcher.

**Results:**

The analysis generated that personal resources and agency were experienced as the overarching facilitators of recovery, with five themes: (1) *Doing recovery in everyday life*, involving agency in daily life; (2) *Re-evaluating risk*, involving re-evaluating limitations and stress reduction; (3) *Becoming a caregiver*, involving development from being cared for to taking care of others; (4) *Negotiating normality*, involving identity and social inclusion; (5) *Owning and sharing your story*, involving accepting lived experience and overcoming stigma.

**Discussion:**

All participants described themselves as the main facilitators of their own recovery, and treatment as secondary to their efforts. Gradually testing limitations and taking risks, providing social support to others, as well as owning and sharing your story were crucial for promoting long-term recovery in FEP. Clinical implications include supporting service users’ agency with strength- based interventions and shared-decision making, as well as refining psychoeducation on stress reduction in a long-term perspective.

**Supplementary information:**

The online version contains supplementary material available at 10.1186/s12888-025-06681-y.

## Background

Psychotic disorders have been thoroughly investigated by outside observers since psychiatry’s infancy [1, 2]. Measuring objective outcomes of symptom remission and adequate functioning is often called clinical recovery. Personal recovery, the subjective process of finding meaning and a good life regardless of symptoms, is a more recent research focus, although it is often reported as more important by people with psychotic disorders [3–5]. Personal recovery has traditionally been researched with qualitative interviews to learn about the process of “living as well as possible” [3, p. 38] from people with lived experience, called experts by experience [3, 6].

Recent studies have called for more research on personal recovery in psychotic disorders, specifically [7, 8]. Psychosis has been referred to as an indescribable experience because it can profoundly alter one’s sense of self, as well as provide new insight [9]. Recovery occurs in a social context [10] and stigmatization, social exclusion, and low socioeconomic resources are often reported as barriers to recovery for people with lived experience of psychosis [8, 11, 12]. Furthermore, psychosis is experienced in several disorders. However, personal recovery is rarely researched across both non-affective (schizophrenia spectrum) and affective psychotic disorders (bipolar and major depressive disorders) [6], although many first-episode psychosis (FEP) cohort studies include both [13, 14]. As such, the recovery process in psychotic disorders, broadly, might entail different aspects than those captured by general personal recovery frameworks.

The CHIME acronym for five key personal recovery processes (connectedness; hope; identity; meaning; and empowerment) is the most well-known and utilized theoretical representation of personal recovery [15]. Nonetheless, one study questioned the utility of CHIME because it describes positive developmental processes that are unspecific to mental illness [16]. In addition, the constructs of personal recovery, such as meaning, are often described as both defining and promoting recovery at once [17]. Additionally, studies have primarily been concerned with defining the recovery process [8, 9, 15] and less often on what is seen as important for recovery.

A qualitative review explicitly differentiated between *characteristics* and *facilitators* of personal recovery in psychosis [8]. Core facilitators of social support, agency, hope, environmental resources and recovery-oriented treatment [8] were also found in a quantitative review [18]. Recovery facilitators were further divided into treatment-, illness, individual- and social environment-related factors by a third review [19]. Individual-related factors, including hope and empowerment, were the most researched and highly related to the subjective process of personal recovery [19]. Therefore, learning from people with lived experience about more specific recovery facilitators could deepen the understanding of what aids recovery in psychosis.

More research on how this process develops over time is needed [18]. Recovery evolves in stages [3, 20]. *Long-term recovery* might therefore involve different processes than the more immediate concerns of adjustment in early recovery [20–22]. Long-term recovery is not frequently explored qualitatively, because studies often include informants with unknown or varying stages of illness and recovery [8, 9, 23, 24], or early recovery in FEP [20, 25].

Few qualitative studies have purposefully recruited informants from longitudinal FEP studies of 10 years or more to explore long-term recovery. Among these studies, two focused on facilitators of clinical recovery [26, 27] and two on characterizing long-term personal recovery, as well as treatment-related factors [22, 28]. Qualitative research on facilitators of long-term recovery from the perspective of participants in longitudinal FEP follow-up studies are therefore lacking and could have clear clinical implications.

The aim of this study is therefore to explore what people with lived experience of psychosis highlight as important for their long-term recovery.

## Methods

### Context of the study

This qualitative study is part of a mixed methods project on long-term recovery in FEP. We have previously reported on the rate and definition of clinical recovery [29] and the rate of personal recovery and other positive outcomes [4] at 10-year follow-up.

Participants in this interview study were all part of the completed TOP 10-year [30] (Thematically Organized Psychosis, TOP-10) and TIPS 20-year [14] (The Treatment and Intervention in Psychosis, TIPS-20) follow-ups of two catchment area-based FEP studies in Oslo, Norway. In both studies, inclusion criteria included meeting DSM-IV diagnostic criteria for a psychotic disorder (non-affective and affective psychosis, with differences in included diagnoses between TOP and TIPS), age 18–65 years, being clinically stable and able to provide informed consent for participation. Additionally, adequate Scandinavian language skills (see cited studies for further information on TOP/TIPS).

Both cohorts were recruited from the majority of in- and outpatient mental health services in the urban Oslo area, with the catchment area covering about 90% of the Oslo population. The TOP study cohort was recruited between 2004 and 2012 within first year of adequate treatment for FEP (defined as hospitalization and or medication). The TOP 10-year follow-up was completed between 2015 and 2021 with 169 participants at follow-up. The TIPS study cohort was recruited between 1997 and 2001 within a year after FEP. The TIPS 20-year follow-up was completed between 2021 and 2022, with 20 participants at follow-up. Demographic and clinical assessments were completed at follow-up by trained psychiatrists or psychologists. All participants have received catchment area based publicly funded treatment in specialized mental health care and welfare benefits in the Norwegian welfare system.

This current study was pre-registered in the Open Science Framework for increased transparency (see https://osf.io/pmqv6/ for original study plan, aim, sampling strategy, interview format and analytical practice). The methodology was slightly altered from pre-registration due to sampling constraints during the COVID-19 pandemic, as well adjusted based on findings during the analysis, which are described below in *2.5 Analytical process*.

### Participants

This interview study included eleven participants from TOP-10 and 9 from TIPS-20. The participants were purposefully sampled [31] as described below. In the follow-up period (2019–2022) eligible participants were reported on by clinical assessors and consequently invited to participate in a qualitative study. We recruited participants from both TOP-10 and TIPS-20 as they have received treatment for a first episode of a psychotic disorder in the same catchment area 10 years apart. Additionally, their age range could further elucidate the long-term recovery process. We chose to interview people in recovery to address the research aim of exploring what has been important for long-term recovery.

From the TOP-10 and TIPS-20 studies we therefore purposefully sampled interview participants on the basis of their long-term recovery, which we were aware of from their clinical assessments in the larger follow-up studies. This included both personal and/or clinical recovery by criteria utilized in the previous quantitative papers [4, 29] (see Table 1 for definitions of recovery) and by the participants’ own definition (personally defined recovery) [32]. See also Åsbø et al. (2022) [29] and Simonsen et al. (2024) [4] for demographic and clinical information in the TOP-10 study total participant group.

We attempted to sample for diversity on a broad range of identity markers to allow for representation of different voices and perspectives [33], as well as heterogeneity to reflect the larger TOP/TIPS samples. We therefore sampled for variation in gender, age, time since first episode (TOP-10 and TIPS-20) diagnostic group (schizophrenia spectrum and bipolar spectrum disorders with psychosis), recovery type (personal and/or clinical), employment/disability status and ethnic background. Participants in this study were representative of the two larger cohorts on most characteristics, although somewhat less ethnically diverse.

Sampling was concluded at 20 participants when information power was considered sufficient to adequately answer the broad study aim, inclusion of both TOP and TIPS participants, as well as the use of cross-case analysis [34]. Of those asked to participate, only one declined. All participants were clinically stable (psychotic and affective) at time of interview.

**Table 1:** Definitions of recovery

Type of recovery	Definition
Clinical recovery	Psychotic and affective symptom remission and adequate functioning for at least 12 months duration. Psychotic symptomatic remission was defined according to the RSWG (Recovery in Schizophrenia working group) [35] international consensus definition with scores equal to or below 3 on the following PANSS^*a*^ items at time of follow-up: positive symptoms (P1-delusions, G9-unusual thought content, P3-hallucinations), dis- organized symptoms (P2-conceptual disorganization, G5-mannerisms/posturing), and negative symptoms (N1-blunted affect, N4-social withdrawal, N6-lack of spontaneity). Discontinuation of medication is not a requirement of symptomatic remission in the consensus definition. Affective symptomatic remission was defined as an IDS-C^*b*^ score below 14, CDSS^*c*^-score below 7 and YMRS^*d*^- score below 8, as well as not meeting criteria for a current affective episode according to SCID-1^*e*^ at follow-up. Adequate functioning was defined as part-time (≥ 40%) work or study, or comparable functioning, independent living and having a close friend/confidant [29].
Personal recovery	Defined in accordance with CHIME [36]. Operationalized as a score above ≥ 45 on the 15-item version of the Questionnaire about the process of recovery (QPR) [4].
Personally defined recovery [32]	Asked participants: “Do you consider yourself in recovery?” and “What does recovery mean to you?” Note: Instead of the English word *recovery*, we used the Norwegian word “*bedring”* in interviews, which roughly can be translated to “ongoing improvement” and describes recovery as a process. We chose to not use “*recovery”* because it is not in everyday speech and may feel foreign, or participants might specifically associate it with recovery-oriented services and the Norwegian recovery movement.

a PANSS, Positive and Negative Symptom Scale

b IDS-C, The Inventory for Depressive Symptomatology, Clinician-rated

c CDSS, Calgary Depression Scale for Schizophrenia

d YMRS, Young Mania Rating Scale

e Structural Clinical Interview for DSM-IV Axis I disorders

### Researchers

The core research-team of GÅ, CS, HH and SHK carried out the cross-case analysis. The team was homogenous as white, primarily female, Norwegians situated in the knowledge-field of psychology, but heterogeneous in age, background, and level of expertise. CS is a clinical psychologist and senior researcher with experience from early intervention psychosis services and psychosis research. HH is a clinical psychologist and professor emerita with in-depth expertise in qualitative methodology and culture- and developmental psychology. GÅ is a clinical psychologist and PhD candidate with experience from psychosis services. SHK is a peer-researcher with expertise from lived experience and advocacy work. He was involved in all stages of the study, provided feedback on the study planning, and interviewed participants. Additionally, he participated in all analytical meetings where he facilitated in-depth discussions about lived experience and the partial perspectives of researchers/clinicians [37, 38].

### Interviews

Interviews were completed in Norwegian between 2021 and 2022. Eight were conducted digitally via secure video software due to covid-19 restrictions and one due to geographical location. Interviews lasted between 1:30–2:05 h and were audio-recorded and transcribed, quotes in this paper were translated to English. TSD (Services for Sensitive Data), developed and operated by the IT Department (USIT) at the University of Oslo, was used for data storage. All participants provided written informed consent at follow-up. Those invited to this qualitative study received additional information about the study purpose and topic.

The interviews were based on the Life Mode Interview, a conversational interview format focused on everyday life [39, 40]. In the Life Mode interview the participant is asked to recount the concrete events of the day before with follow-up questions about typical routine, inter-personal relationships and how this life mode developed. As opposed to a structured interview, the participant therefore largely guides the conversation.

In this study, we asked relevant follow-up questions about recovery and facilitators based on an interview guide (see in supplement). In all interviews, the conversation quickly branched out from the events of yesterday to include reflections around recovery journey, service experiences and relationships that had supported or hindered recovery. In an example of how relevant topics typically occurred in the interviews, one participant immediately demonstrated what had been important for her recovery when she described her morning routine of yoga and spiritual practices. As recovery is a process of development occurring in daily life [41], we found the Life Mode interview to be especially suited to explore what had facilitated the participants’ recovery as they experience it [42]. The interview was concluded with a more general question: “What does recovery mean to you?” to prompt for the participants’ characterization of their personal definition of recovery [32].

### Analytical process

The analysis was a team-based thematic analysis informed by reflexive thematic analysis (TA) [43, 44], based in a contextualist framework [33]. This epistemological framework is also referred as perspectivism [45]. From this framework the researchers viewed the knowledge generated from the analysis as context-specific and that the participants held the truth about their recovery process. Furthermore, that this truth could likely not be fully accessed because the researchers’ interpretation of the interviews were influenced by our own partial perspectives [38, 46]. However, our situatedness and the experiences we brought into the analysis were not viewed as biases that could be bracketed with reflexivity, but something that were a part of the knowledge creation. Therefore, we did not refer to the themes as something that passively emerged from the material. Rather, that results were generated as they were seen as a co-creation between participants and interviewers/researchers. Moreover, within a contextualist position the goal of the team-based analysis was not to come to an inter-rater agreement, but to gather several perspectives, especially including that of a stakeholder in the peer researcher [45, 46]. By including differing voices in the analysis we aimed to come closer to a “completeness of perspective” [33] or a more comprehensive view of the participants’ recovery process.

The analytical process was carried out systematically in six steps (see below). The analysis was largely inductive, with a focus on the participants’ life experiences, but the creation of themes was also deductive and informed by theoretical concepts. This team-based TA approach was chosen to increase rigor by allowing for reflexivity around how our context, various types of experiences and expertise informed our interpretation. Additionally, similar team-based approaches have been was utilized in qualitative studies on clinical recovery in FEP [27] and personal recovery in bipolar disorder [47]. TA is suitable for larger datasets where the aim is to capture heterogeneity and social context [48]. The analysis generated five themes, where a theme is understood as: “capturing a core idea or meaning, and the telling of an interpretative story about it.” [49, p. 22].

1. GÅ, CS, HH, SHK separately read each interview as they were transcribed and then met for regular 3-hour research meetings over the course of a year, where each team-member freely shared their first impressions of one interview at a time. Discussions also centered on reflexivity and how our experiences related to the participants and shaped our interpretations, where particularly our different ages and life stages were influential. In addition, the larger follow-up studies, and the social context in which this knowledge was created was frequently highlighted. GÅ created analytical memos for each participant based on these discussions, as well as organized data relevant to the research aim in an Excel spreadsheet. GÅ also utilized NVivo 12 Pro software to create initial codes based on memos.
2. After the first initial analytical meetings, the research process developed from analysis within interviews to comparing across interviews on various characteristics. The most salient tendency in this stage was how the participants consistently described themselves as the central agents in their recovery and that they understood their own agency and effort as the most important facilitator. Services appeared to have more indirectly influenced recovery in the long-term. We therefore chose to focus the analysis on individual- and social environment related facilitators of recovery, or personal resources, and utilize the substantial material concerning service user experiences for a later paper.
3. When all 20 interviews were discussed separately, GÅ returned to the dataset as a whole to further condense meaning by collating codes and memos into topic groups or “potential themes” as described in Braun and Clarke (2006) [44]. Topic groups were based on the most salient similarities/dissimilarities between participant accounts and relevant theory, for instance: “stigma”. Potential themes were presented to the whole team for review.
4. GÅ and SHK then re-read interviews within each potential theme/topic group to scrutinize, deepen and add nuance. Interpretations were shared in meetings with CS for further discussion.
5. Based on this process, GÅ then generated five themes of central processes promoting long-term recovery in FEP. GÅ reviewed the themes at the level of each participant, topic group and data material as a whole to ensure that each theme captured the participants’ narratives.
6. The five themes were presented to the research-team for further nuance and refinement. Final themes and names were agreed upon by the team.

## Results

*“I’m in a more recovered recovery than before (…) It’s getting better*,* but not in the way I thought it would*,* at all. It’s always about how things aren’t going like I thought they would. Because maybe I have very high expectations or thoughts about how things should be…but let me*,* then…I’m thinking*,* why shouldn’t I expect a lot from life?”* (Female, 20s).

Viktoria is one of the 20 people we interviewed for this study (see Table 2 for demographic and clinical information). She is a young adult who first experienced psychosis 10 years ago. Today, she has her own apartment, a social network, a job she likes and no longer experiences psychosis. From the outside she appears clinically recovered. In her own esteem, she is in the beginning of her personally defined recovery process because she is considering taking *“a leap”* to pursue a long-term career goal she was initially advised against. She is asking both herself and those around her why she shouldn’t expect a lot from her life.

**Table 2:** Demographic and clinical characteristics of study sample (*n* = 20)

	Participants
Demographic	
Gender, female	11
Age (Mdn, range)	46 (28–73)
Norwegian origin	19
In relationship	9
Have children	11
Full-time work/study	8
Part-time work/study and disability pension	2
Retired, aged based pension	3
Full-time disability pension	7
Clinical	
Diagnosis, 10 years	
Schizophrenia spectrum	14
Bipolar spectrum	6
Current use of medication*	14
In current treatment	9

*anti-psychotic/mood-stabilizer

All participants reported that they considered themselves in recovery according to their subjective definition [32]. By criteria utilized in the quantitative studies [4, 29], three were in clinical recovery only, ten in personal recovery only, and seven in both types of recovery. From their clinical assessments it was reported that most participants in clinical recovery had met clinical recovery criteria for the majority of the follow-up period. Furthermore, almost all participants reported to be engaged in a long-term recovery process of their own definition that for most began shortly after their first episode. For some, however, this process had started more recently. Recovery was largely described as an enduring non-linear process of creating a good everyday life. A few participants in long-term clinical recovery described themselves as fully recovered and their lived experience belonging to the past. Over half of the participants defined recovery as being “symptom-free”, “stable”, or “well-functioning”, although few reported that this was sufficient for long-term recovery. Several also defined recovery in line with terms such as “acceptance” or “independence/empowerment”.

**Fig. 1:**
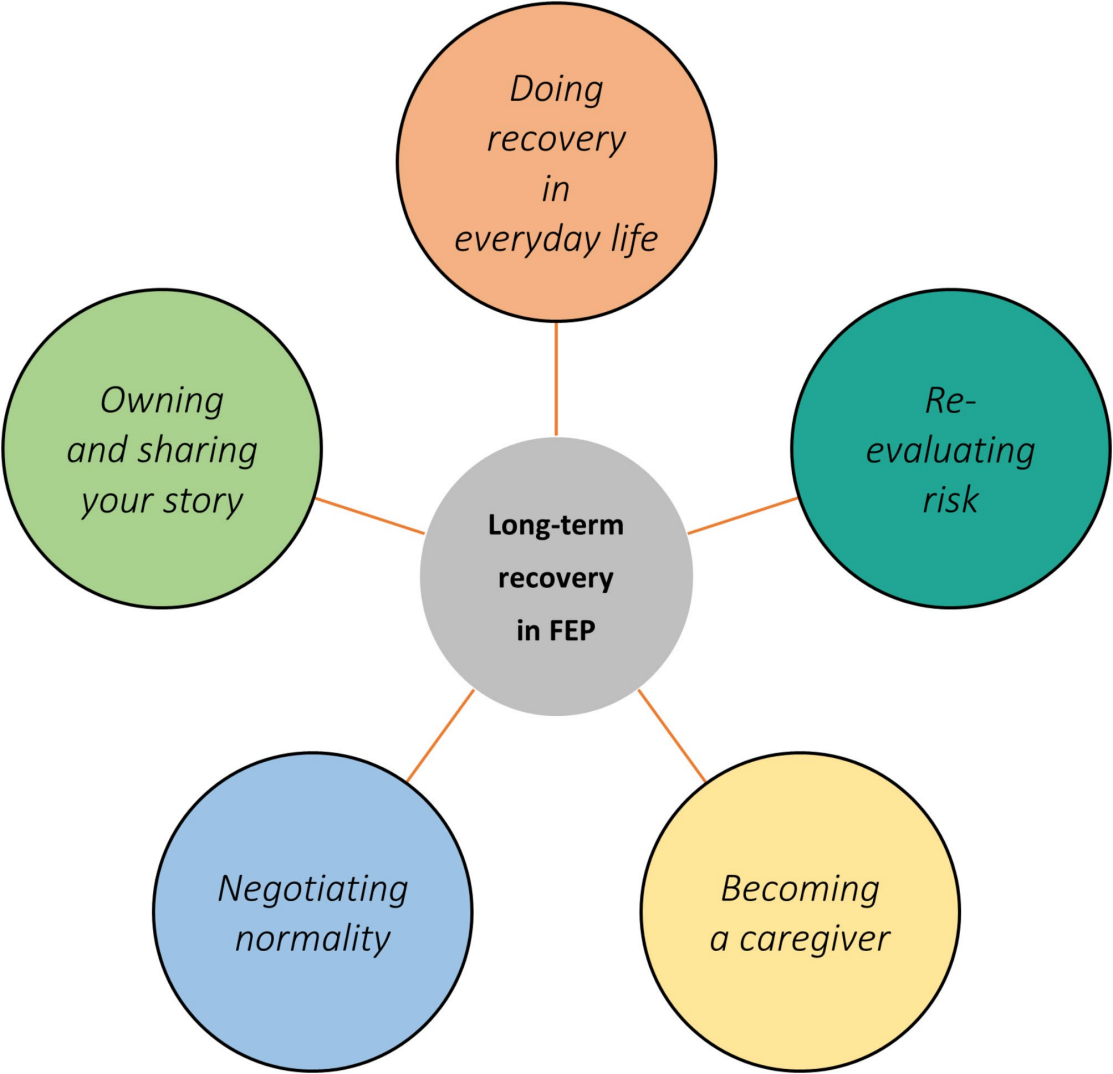
Five themes of facilitators of long-term recovery in FEP

The overarching result was the participants’ personal agency and resources, particularly that they viewed themselves as the main facilitators of long-term recovery. Based on the thematic analysis, we generated five themes (see Fig. 1) of important facilitators of long-term recovery in FEP (see supplementary material for additional representative quotes for each theme). (1) *Doing recovery in everyday life* (2) *Re-evaluating risk*, (3) *Becoming a caregiver*, (4) *Negotiating normality*, and (5) *Owning and sharing your story*.

### Theme 1: Doing recovery in everyday life

This theme illustrates that all participants have utilized their agency to arrange a good life. Furthermore, the participants described several concrete facilitators of recovery that they actively utilized in their everyday lives. These were often formulated as tips for recovery to others with lived experience. As such we named these concrete suggestions *recovery strategies*. The recovery strategies participants employed differed greatly and included: exercise, keeping routines, sleep, stress management, appreciating nature, spirituality, and self-monitoring (See Table 3 for selection of participants’ recovery strategies). However, beyond individual strategies, we found that almost all participants described recovery as something that they were actively *doing* in their day-to-day life.

**Table 3:** Participant recovery strategies

Quotes within theme 1: Doing recovery in everyday life
*“You get a lot of experience from struggling. Also recovery. I mean*,* you get recovery from all the experience you have with struggling(…) Recovery is finding good techniques for mastering life.(…) Less stress*,* try to be in the here and now. And don’t create worries that aren’t here yet*,* worry when the time comes to worry.* (Female, 30s)
*“try to have an active life and do what one is capable of.”* (Female, 50s)
*“I didn’t quit because I know that there are people walking around that are doing ok. It’s not the right strategy for everyone*,* but for me it works to bite down on the shin (idiom) and keep on until things get better.”* (Male, 40s)
*“I’ve always exercised (…) It means a lot for the mind…in my thoughts. It gives me more space and I feel better. Life gets calmer and you’re better equipped to handle thoughts*,* they can just come and go and you can handle it no matter what. Then there’s less stress.”* (Female, 40s)
*“I have a planner*,* and I try to schedule the week (…) I try to have an activity every day. Changing tires*,* emptying the dishwasher*,* vacuum*,* clean the bathroom. And find a rhythm*,* structure*,* structure*,* structure.”* (Male, 30s)
*“On with your shoes*,* on with your jacket*,* go out for a walk… What’s been the most helpful for the brain is to go out for a walk.”* (Female, 60s)
*“When I meditate and find that space*,* when I focus on what makes me happy…then the racing thoughts don’t start as easily(…) if you can create that space so the shame lets go then it’s not so bad to be in pain*,* then it’s not so bad that you can’t do everything you wanted.”* (Female, 50s)
*“I’ve started to notice what’s around me. The joy I feel on a fall morning is so deep that it’s hard to explain.”* (Male, 40s)
*“Recovery is that I can get up in the morning(…) It means that you’re keeping up with something. That you can manage a 50% job*,* or some program*,* or that you’re going somewhere and taking a class or whatever. That you’re headed somewhere*,* in a way. (…) I know I can work more than 50%*,* but I think 50 is nice for getting some peace and for my concentration…and that I need some rest between activities and because I still need to be considerate to myself every day.”* (Female, 20s)
*“The first medicine is to get enough sleep*,* right. To calm the mind and get some rest.”* (Male, 40s)
*“you have to let yourself collapse. If you have a barn that’s rotten through to the foundation you actually have to tear that shit down and build a new one.”* (Male, 30s)

We learned the most about everyday recovery from participants that were at home during the day. Dorthe had an engaging career when she first experienced psychosis. Her recovery process has entailed finding meaning outside employment, accepting herself and actively collecting what brings her joy. She for instance mentioned watching daytime TV, which she was initially ashamed of. Now she allows herself to enjoy TV and actively uses what she watches to be up to date on current events and for conversation starters:

*“it’s about seeing what bringsyourecovery. And then follow those tracks. Grab a hold of what is positive and people that are good for you.”* (Female, 50s).

Like Dorthe, underlying all the participants’ accounts were their agency and ownership over their recovery. This recovery largely occurred in their everyday lives.

### Theme 2: Re-evaluating risk

This theme captures that in long-term recovery several participants have taken positive risks to follow what they expected out of life, but how far along they were in this process differed. Although most participants appeared content with their lives, about half seemed more future-oriented and less concerned with risk of relapse and stress in search of what brought them meaning. William described his creative career as a “*meaning hook*” that was “*something to hold on to*” after his first hospitalization. This career is also unpredictable and highly stressful:

*“I am working on four different projects at the same time (…) it’s probably because I have crazy expectations of myself*,* and I always have.”* (Male, 30s).

William stated that he has always been driven. However, several participants demonstrated the temporal aspect of this theme as they had more recently begun to reconsider their limitations, a term several participants utilized. Dag previously met all criteria for clinical recovery. However, he expressed that he was unhappy and, in his own words, drifting along on an “*inflatable pink flamingo*” of life because he thought he should “*accept limitations”*. He has recently left his stable employment and living situation to pursue a more subjectively meaningful path and is, by his own admission, finally in a personal recovery process:

*“It’s been a pretty drastic improvement…I’ve actually begun to look at myself and all the unhealthy coping mechanisms through the years*.*”* (Male, 30s).

A small group of participants stated that they had some goals or dreams they never followed, but that they are now considering taking a leap like Victoria who was introduced in the beginning. Like her, these participants expressed that they feared experiencing stress and related relapse, and that they did not trust their own judgement about minor life choices. Although she is happy with her life, Dorthe from the above theme is considering returning to the work force, but is unsure because her provider cautioned her against it in early recovery:

*“I have been warned by my psychiatrist to think like that (…) I could get sick again if I entered the job market. It says so in my papers at least. In my discharge papers it said that I should avoid stress*,* so I would have a better prognosis (…) I can’t handle stress”* (Female, 50s).

For several participants, it seemed that an important facet of long-term recovery was to develop from living a more limited life focused on avoiding stress and relapse, to gradually testing their limitations and engage in some risk to live life to the fullest.

### Theme 3: Becoming a caregiver

This theme illustrates a development in how participants related to their social support network. Many participants had been supported by family in early recovery, and most had a social network of people who were important to them, such as parents, partners, and friends. However, when reflecting on the role of social support in long-term recovery, most participants highlighted their role as a parent, partner or friend and support to others. Almost all participants had developed from being primarily cared for, to now also becoming caregivers for others through for instance having children, engaging in social justice or volunteer work.

Participant Iben has an active social life, but when asked about the role of her friends in recovery, she responded: *“friends are not therapists”*. Instead, what appeared important for her long-term recovery was her self-professed “*care-gene*” and to take care of her aging parents who took care of her during her first episode many years ago. Many participants discussed that having children to care for also helped them take better care of themselves and provided a sense of purpose.

For several participants, offering care and support to others was therefore a turning point in their recovery. Emil had been taken care of by services and family for a long time. He previously felt shame for receiving disability pension, but described that helping friends and family made him feel more included:

*“Be a resource in your community*,* be a brother or sister*,* father or mother (…) I mean something to other people*,* I have value…I’m not on the outside”* (Male, 30s).

In addition to providing meaning and purpose, taking care of others appeared to have granted many an identity beyond that of a service-user or patient. This identity and positive role appeared to demonstrate for many participants that they were valuable citizens.

### Theme 4: Negotiating normality

This theme captures an internal diversity in how participants viewed their identity in relation to their social context, and what kind of lives they wished to live. Over half of the participants reflected on what is ordinary or *normal*, a word brought up by several. Most of these participants expressed that they wished to be seen as normal and catch up to peers in developmental milestones after their experiences with psychosis or mania left them feeling excluded. Magnus struggled as a teenager, reported that he belonged to a rough crowd and eventually dropped out of high school. He expressed feeling behind his peers after spending some years in treatment and work-placement programs. As an adult, he has worked hard to achieve a life he described as “normal”, but due to his past he is aware that there are many ways to live life and many paths to recovery:

*“an important part of it is to be*,* maybe it’s a bad word in psychiatry*,* but to be ‘normal’*,* seriously…I just want to be very average*,* actually. I just want an ordinary steady job*,* and a family and a house and a garden and a car and…(…) but if you want to be a bohemian*,* musician*,* that’s also a way to be better*.*”* (Male, 20s).

This theme is titled *negotiating* normality because we also noticed a smaller group of participants who seemed to value a more alternative lifestyle and reject normality. For these participants figuring out who they were and how they wished to live their lives was a large part of their long-term recovery. One participant defined recovery as: “*freeing yourself from social norms.”* Participant Monica described that she disagreed with how she had frequently felt pushed by treatment providers to conform or live a certain way to be seen as recovered:

*“Who gets to decide who has a good life*,* you know?”* (Female, 50s).

For Geir, reflecting on his identity and what is “normal” or not was a longer process. He became concerned with how he was perceived by others after his first psychotic episode, but described that it has been important for his recovery to worry less about what others think:

*“After a while*,* I have stopped trying to figure out what is normal or not*,* people are just different*.*”* (Male, 40s).

From participant accounts, concerns about normality or being ordinary appeared linked to social inclusion and a desire to belong. Participant Liv has struggled with her mental health for most of her adult life and has often felt that she did not belong anywhere. She has made great changes to her life in recent years and has discovered how important social inclusion is for her recovery. Liv stated that filling out a questionnaire-item about belonging during the follow-up assessment made her realize that she finally felt included:

*“I was so happy about that question: do you feel like part of society? Yes! (elated). Because I didn’t before…It’s lovely to be an ordinary person in society!”* (Female, 60s).

For the participants, social inclusion meant many things, including the freedom to express their non-majority culture, emotions, or spirituality without being judged as psychotic or manic. This theme is therefore not just about identity or accepting who you are, but also about the importance of being accepted and included by others for long-term recovery in psychotic disorders.

### Theme 5: Owning and sharing your story

This theme illustrates that almost all participants have created a cohesive narrative about their lived experience that they could share with others, as they did in the interviews themselves. It appeared that openness or sharing their story was crucial for their recovery. Several participants discussed strategic openness, when to be open and what to say to receive support or necessary accommodations, and that being open allowed them to connect to peers with the same experiences. In addition, some reflected on how they welcomed the increased openness about mental illness in society.

Nonetheless, it appeared that a prerequisite of this openness was feeling secure and accepting their experiences, but this was for many hindered by public stigma towards psychotic disorders. Most participants with bipolar disorder seemed to reject stigma or were more concerned with not using their diagnosis to seem more *“interesting”*. However, almost all participants with schizophrenia disliked their diagnostic label and worried that people would judge them if they disclosed it:

*“I’ve never liked that diagnosis*,* that schizophrenia diagnosis (…) I’ve never had a clear sentence for how I’m going to say that I am sick(…)I have never really accepted it. I’ve struggled a lot with that. So*,* there’s something about owning your story when you’re supposed to talk about it*.*”* (Female, 30s).

Participant Lars felt that the label of schizophrenia has affected how he views himself and is viewed by others:

*“To get that label on you is also a burden…before I got that label*,* I wasn’t that sick (…) People get afraid*,* there’s a lot of talk about madness. (…) It’s not easy when you’ve had problems with psychosis and stuff*,* and you’re not able to be honest. Having two things to handle*,* an outer mask and inner turmoil…to let that go is important.”* (Male, 40s).

A few participants with schizophrenia also rejected the stigma against their diagnosis and it seemed to have helped them accept themselves:

*“Why should I care about that* (schizophrenia diagnosis), *I know I’m a good person in society*,* what more can you expect of me?”* (Male, 40s).

As Lars demonstrates, owning and accepting one’s story appears important for recovery, because it is both taxing and isolating to hide who you are.

## Discussion

This qualitative study is the first to explore facilitators of long-term recovery (personal and/or clinical) from the perspective of participants in two follow-up studies 10 and 20 years after treatment start for first-episode schizophrenia and bipolar spectrum disorder. Recovery was described as an enduring process most participants were still engaged in, which allowed for an exploration of the nature of long-term recovery in psychosis. Additionally, the participants’ stories inspire hope as they further illustrate that recovery is an ongoing development where positive changes and growth also occur later in life.

The most striking finding was that all participants highlighted themselves as the greatest facilitators of their recovery, above the role of treatment. Agency, being socially included and overcoming stigma seemed especially important for their recovery, in line with several previous studies on psychosis [8, 15, 18, 19, 23, 24]. The most novel result was that taking positive risks and re-evaluating the need for stress reduction over time [17] appeared as crucial for long-term recovery in psychosis. Furthermore, our finding on the importance of providing social support to others [24] has rarely been reported. This study therefore contributes valuable information to the literature about long-term recovery in psychotic disorders.

Empowerment and agency is a core tenet of the personal recovery perspective [15]. It is worth noting that the importance of agency has been misused to construe recovery as a personal responsibility and not recovering as a personal failing [50–52], also called *responsibilization* [53]. The participants in this study have perhaps had a less challenging context to their development of agency than other service users, as they are all in recovery and have received support from a well-built public health and welfare system.

However, psychosis recovery was traditionally seen as an outcome of successful treatment [54]. As such, there is value in further demonstrating that people who have historically had little agency have also facilitated their own recovery [12]. Furthermore, in line with previous qualitative studies we found that the participants’ recovery process did not primarily occur within specialized mental health services, but in their everyday lives [15, 24, 27, 41, 47, 55].

The importance of balancing stress and accepting limitations to avoid disappointment or relapse is a common qualitative finding in schizophrenia and bipolar disorder recovery [41, 56]. For how long limitations should be accepted is less frequently discussed. We found that a crucial facilitator of long-term recovery in psychosis was gradually re-evaluating limitations and eventually taking some positive risks for a fuller life. However, from participant accounts, many were concerned that following goals would lead to stress, which they had been told to avoid. Stress reduction and management is linked to more favourable outcomes in FEP, and therefore central in early intervention programs [57–60]. Stress reduction is based on the stress-vulnerability model [61]. This model describes how biological vulnerability factors reduce the ability to tolerate stressors (including life stressors, trauma and adverse events). Stress that overwhelms this threshold is then hypothesized to be a causal factor in the development of psychosis [61–63]. Nonetheless, central to this model is also that stress is subjective and not static. Some stressors are positive, social exclusion can also be stressful, and the need for reducing stress might change in long-term recovery. These issues are perhaps less frequently communicated in early psychosis psychoeducation than the importance of avoiding stress.

Relatedly, a personal recovery review [17] found that themes of risk-taking is disappearing in favour of safety in the literature and called for more research on this topic. This review, along with a paper by Felton and others (2018) in The British Journal of Psychiatry [64], discussed the importance of therapeutic risk-taking for recovery. Both papers referred to Patricia Deegan’s influential work on recovery and agency in schizophrenia and her well-known quote about the importance of supporting “the dignity of risk and the right to failure” [64, p. 84].

Social support is one of the most consistently reported facilitators of personal recovery, also in psychosis [8, 15, 18, 19, 24, 65]. Although participants valued their social support network and many had family members who were central in early recovery, when prompted, surprisingly few discussed that social support had a large impact on their recovery currently. This could partially be explained by Norway’s public health system which is less reliant on informal caregivers.

Another explanation for this finding is that almost all participants had over time developed from being taken care of to taking care of others. This identity and social role [42] appeared to give them both a sense of meaning and citizenship, which remarkably few studies discuss [24, 66]. Even within research on peer-support, results are mostly focused on the value of receiving it [8, 15]. The beneficial aspects of pro-social behaviour are unsurprising as giving to others is one of the “five ways to wellbeing” in general wellbeing research [67]. However, this is rarely reported in relation to people with psychotic disorders, who are often viewed as passive recipients of support. As such, the importance of providing support should be explored in future research and implemented in treatment, through for instance facilitating volunteering or advocacy work.

Regaining social status and being so-called normal or ordinary after a first episode of psychosis or mania was thematized by many participants. The desire for normality [22, 54, 56] and the importance of recovering identity and a sense of self [68, 69] appears as a core concern for people with lived experience of psychosis [24, 68, 70]. For other participants, it seemed important for recovery to find a less conventional lifestyle that suited them, although they were equally concerned with feeling included.

The crucial role of social inclusion is a longstanding focus of psychiatry [71] and found in several studies on recovery in psychosis [8, 22, 24, 72]. However, according to Davidson et al. (2001), social inclusion of people with psychotic disorders should not be dependent on meeting the “ideal of normality” [72]. Similarly, “making people independent and normal” instead of focusing on citizenship and the rights to participate in all social institutions has been named an abuse of recovery [52]. As such, to promote recovery for people with psychotic disorders, social participation should be aided through for instance employment support and social activity. However, to ensure social inclusion “communities that can accommodate all of us” are also needed [10].

Accepting and changing one’s narratives from failure to pride in accomplishments appeared as an important development in long-term recovery. This seemed to further facilitate the safety to be open about lived experience, or sharing one’s story which is also crucial for recovery. Hiding a concealable stigmatized identity, such as sexual orientation or mental illness, is known to be psychologically damaging and isolating [73, 74]. Although personally overcoming stigma is central to recovery according to the CHIME framework [15], this process is particularly challenging for many people with psychotic disorders due to significant stigma. Therefore, addressing public and systemic stigma is essential to aid recovery for people with psychotic disorders [11].

### Clinical implications

The first clinical implication of this study is that service users’ personal resources should be encouraged in treatment through strengths-based interventions and shared decision-making. Secondly, employment or other subjective recovery goals are equally important to symptom alleviation. Particularly, facilitating or encouraging caregiving such as volunteering, or peer support work could aid recovery. Thirdly, discussing stigma appears helpful based on participants’ concerns. Lastly, the most important clinical implication is that services, particularly early intervention, could nuance psychoeducation about stress reduction to include that the ability to manage stress will change over time and that some stressors are positive. Relaying this, and the importance of therapeutic risk-taking to both service-users and their caregivers could be helpful. In addition, services could view recovery as a long-term process that will continue to develop after the person is discharged. The messages people receive in treatment could come with the information that they should be continually re-evaluated.

### Methodological reflections: strengths and limitations

The primary strength of this study is the participant group sampled from two representative long-term FEP follow-up studies that include both schizophrenia and bipolar spectrum disorders. This strength enhances transferability to other broad FEP cohorts. Furthermore, we interviewed participants in long-term recovery both by criteria and personal definition who could reflect on their process. This is another substantial strength of our study that might heighten the resonance of results to people with lived experience of psychosis. Working as a research team with a peer-researcher as an integrated member strengthened rigor, reflexivity, as well as clinical relevance of the results. Furthermore, the scope of the more open interview format, based on the Life Mode interview [39] was more comprehensive than a semi-structured interview and granted access to rich recovery-experiences [42]. This study’s limitations are also tied to context [33]. Sampling from an ethnically homogenous cohort partaking in psychiatric research could leave out other crucial experiences and more critical perspectives on psychosis and recovery [75]. The COVID-19 pandemic impacted the participants’ current life, but few discussed that it affected their recovery. Utilizing a Norwegian translation of personal recovery (“bedring”) also has benefits and challenges. The main benefit is that “bedring” is a familiar word in everyday language that might better resonate with the participants’ personal process than a foreign construct (“recovery”). However, using a local term can impact transferability to other recovery studies, a dilemma non-English speaking qualitative studies on recovery should perhaps further discuss and research. Furthermore, this study was focused on mostly individual-related factors of recovery, exploring other recovery facilitators such as treatment, as well as barriers, are important focus areas for future research.

### Conclusion

The main contributions of this interview study on long-term recovery in psychosis include the participants’ own role in their recovery. The most novel findings were the importance of taking positive risks to follow expectations of life, being a caregiver for others, and owning and sharing your story. Early psychosis or bipolar intervention services could reflect these findings to support service users’ strengths and subjective recovery. Moreover, to include psychoeducation on the fluctuating need for stress reduction and limitation in a longer-term perspective.

## Electronic supplementary material

Below is the link to the electronic supplementary material.

Supplementary Material 1

## Data Availability

This study was pre-registered in the Open Science Framework: https://osf.io/pmqv6/. The datasets generated and analyzed during the current study are available from the corresponding author on reasonable request.
