# Supplementary material for: “Why shouldn’t I expect a lot from life?” – a qualitative study of what facilitates long-term recovery in first-episode psychosis
Source: BMC Psychiatry. 2025 Apr 28;25:423. doi: 10.1186/s12888-025-06681-y (PMC12036267; doi:10.1186/s12888-025-06681-y)
Supplement: Supplementary file 1 — Supplementary Material 1 [file 12888_2025_6681_MOESM1_ESM.docx]

# Supplementary material: “Why shouldn’t I expect a lot from life?” – a qualitative study of what facilitates long-term recovery in first-episode psychosis

Gina Åsbø*^1,2,3^, Hanne Haavind^3^, Sindre Hembre Kruse^2^, Kristin Fjelnseth Wold^1,4^, Wenche ten Velden Hegelstad^5,6^, Kristin Lie Romm^2,1^, Mike Slade^7,8^, Torill Ueland^1,3^, Ingrid Melle^1,4^, Carmen Simonsen^2,1,3^

^1^ Section for Clinical Psychosis Research, Department of Research and Innovation, Division of Mental Health and Addiction, Oslo University Hospital, Oslo, Norway

^2^ Early Intervention in Psychosis Advisory Unit for Southeast Norway, Division of Mental Health and Addiction, Oslo University Hospital, Oslo, Norway

^3^ Department of Psychology, Faculty of Social Sciences, University of Oslo, Oslo, Norway

^4^ Institute of Clinical Medicine, University of Oslo, Oslo, Norway

^5^ TIPS – Centre for Clinical Research in Psychosis, Stavanger University Hospital, Stavanger, Norway

^6^ Faculty of Social Sciences, University of Stavanger, Norway

^7^ School of Health Sciences, Institute of Mental Health, University of Nottingham, Nottingham, UK

^8^ Faculty of Nursing and Health Sciences, Health and Community Participation Division, Nord University, Namsos, Norway

*Corresponding author:

email: [gina.asbo@psykologi.uio.no](mailto:gina.asbo@psykologi.uio.no)

ORCID: 0000-0003-3135-3522

## Interview Guide

- **Interview method**:
  - Life Mode interview (1,2):
    - Example of questions asked in a typical Life Mode interview: “can you walk me through your daily routine, for instance, where did you wake up yesterday? and what did you do after you woke up?” Adding questions about living situation, social circle, activities, typical routine and uncommon events
    - Connecting questions on everyday life to development and process, for instance: “how long have you..?”, “has it always been like this?” “this seemed like an important event to you?” “what has changed?” “what made this possible?

Specific questions related to recovery will be integrated into the Life Mode interview. Participants will be asked to deliberate when they bring up relevant topics to research aim, or if no such topics are discussed by the participants, questions below can be utilized.

|  | **Interview questions**  Main question:   - Potential follow-up question, if necessary |
| --- | --- |
| **How is the recovery process described?** | From your own understanding of what recovery is, do you consider yourself recovered?”   - How do you know? - When did you know?   How would you describe your recovery process?   - Has anything been particularly challenging? - Have things turned out differently than you imagined? - What have you learned from the recovery process? - How happy are you with where you are now? Can anything improve?   How do you picture yourself and your life in the future? |
| **What has been important for recovery?** | What has been important for your recovery?  How have you yourself contributed to your recovery?   - What do you think about this now? Would you have done anything differently? - Has this changed how you see yourself, and how?   How have the people around you contributed to your recovery?   - What do you think about this now? Could they have done anything differently?   How have you experienced the mental health system and how has it contributed to your recovery?   - Has it hindered your recovery in some way? - Has any type of treatment been more helpful than others? Which? - How could the treatment further aid recovery? - Did you receive enough treatment? |
| **How is recovery defined?** | What does recovery mean to you? |
|  | Is there anything else you would like to add? |

## Representative quotes for each theme

| Themes  Subgroups | Quotes |
| --- | --- |
| 1. Doing recovery in everyday life | |
| Participant recovery strategies | *“You get a lot of experience from struggling. Also recovery. I mean, you get recovery from all the experience you have with struggling(...) Recovery is finding good techniques for mastering life.(…) Less stress, try to be in the here and now. And don’t create worries that aren’t here yet, worry when the time comes to worry.* (Female, 30s)  *“try to have an active life and do what one is capable of.”* (Female, 50s)  *“I didn’t quit because I know that there are people walking around that are doing ok. It’s not the right strategy for everyone, but for me it works to bite down on the shin (idiom) and keep on until things get better.”* (Male, 40s)  *“I’ve always exercised (…) It means a lot for the mind…in my thoughts. It gives me more space and I feel better. Life gets calmer and you’re better equipped to handle thoughts, they can just come and go and you can handle it no matter what. Then there’s less stress.”* (Female, 40s)  *“I have a planner, and I try to schedule the week (…) I try to have an activity every day. Changing tires, emptying the dishwasher, vacuum, clean the bathroom. And find a rhythm, structure, structure, structure.”* (Male, 30s)  *“On with your shoes, on with your jacket, go out for a walk… What’s been the most helpful for the brain is to go out for a walk.”* (Female, 60s)  *“When I meditate and find that space, when I focus on what makes me happy…then the racing thoughts don’t start as easily(…) if you can create that space so the shame lets go then it’s not so bad to be in pain, then it’s not so bad that you can’t do everything you wanted.”* (Female, 50s)  *“I’ve started to notice what’s around me. The joy I feel on a fall morning is so deep that it’s hard to explain.”* (Male, 40s)  *“Recovery is that I can get up in the morning(…) It means that you’re keeping up with something. That you can manage a 50% job, or some program, or that you’re going somewhere and taking a class or whatever. That you’re headed somewhere, in a way. (…) I know I can work more than 50%, but I think 50 is nice for getting some peace and for my concentration…and that I need some rest between activities and because I still need to be considerate to myself every day.”* (Female, 20s)  *“The first medicine is to get enough sleep, right. To calm the mind and get some rest.”* (Male, 40s)  *“you have to let yourself collapse. If you have a barn that’s rotten through to the foundation you actually have to tear that shit down and build a new one.”* (Male, 30s) |
| Agency | *“medication and treatment alone won’t help, you have to make the most out of the situation also(…)we have to take responsibility for our own health”* (Female, 50s)  *“oh my God, what haven’t I tried (laughs)? I have done a lot* (about recovery)*.”* (Female, 40s)  *“(recovery is to) make my own decisions and my own choices in everyday life.”* *”for many years I didn’t drive, I was just a passenger.”* (Female, 40s)  *“taking the reins of my life.”* (Female, 60s)  *“Since before I entered psychiatric treatment I’ve been used to treating myself.”* (Male, 30s) |
| 2. Re-evaluating risk and not accepting limitations | |
| Messages of limitation and stress-reduction | *“it’s just like they think I should live much narrower, narrow in my life and just live in a sphere that’s ok for me and that I can handle.”* (Female, 20s)  *“That you find something that matches your vulnerability, where you can control it without too much stress and things I’m vulnerable to. I’m vulnerable to stress.”* (Male, 30s) |
| Not accepting limitations | *“accept limitations(…)just lay back and accept what is happening to you and not really have any agency in your own life.(…)Don’t accept other people’s criteria for what a good life is to you.”* (Male, 30s)  *“I can understand that everyone else has their limits, but I’ve always thought of myself as superwoman and that I can do everything.”*  (Female, 30s)  *“People* (with serious mental illness) *often get stuck in a kind of immaturity and limitation in life”* (Female, 40s) |
| 3. Becoming a caregiver | |
| Role as a caregiver | *“I’ve been useful, and that has probably been important”* (Female, 50s)  *“I have the opportunity to contribute to society in a productive way, as a friend and an individual in society… Use the resources you have, get joy from extending yourself, teach others.” (Male, 30s)*  (psychosis has provided) *“Some wiseness, or understanding that when people get sick…it doesn’t have to be that bad.”*  *“It’s usually been me that have had to be there…because I think you should in a way. And when no one else does, I have to.”* (Female, 70s) |
| Caring for and about others is helpful to recovery | *“The kids are more outgoing than other people…that opens some space for me to be myself.”* (Female, 40s)  *“I had to get it together, so I wasn’t completely fragmented”* (When having children) (Male, 40s)  *“Now that I am more mature in a way, and see more, I’m more humble about my own situation. Maybe I can make others happy, help other people a bit, because I have also read something about that if you are very depressed you should help other people, do something for other people.”*  (Female, 60s) |
| 4. Negotiating normality | |
| Identity | *“What’s hard is that expectation to be ordinary or to conform.”* (Male, 30s)  *“The traps that hinder you in reaching your goals are all these expectations about how you should live, that I think many people get stuck in and just think, you have to do it because you should. And then they all sit there with a life that they in many ways don’t want.”* (Male, 30s)  *«I wouldn’t be happy with a 9 to 5…but do you have to be so fucking happy, though?”* (Male, 30s) |
| Social exclusion/inclusion | *“a break with reality and my self disintegrated…I did not have real connections to the people around me”* (about psychosis)*.* (Male, 30s)  *“I have said that I’ve been psychotic and taken medications (…)but I haven’t told many about the diagnosis(…)I told a few friends and the relationships soured after.” (…)* (Female, 50s)  *“You notice a reaction from society to having some unusual thoughts and different references than others”* (Male, 40s)  *“To simply experience that you’re on the inside, as a part of the others…to be like everyone else, that’s really what humans want.(…)To have good friends, function in a context as a part of society in a community, even though I’m on disability(…) Then the healing process will be much quicker than if one falls completely on the outside and have to work from there.”* (Male, 30s) |
| 5. Owning and sharing your story | |
| Openness about lived experience | *“I haven’t been so open, because you meet different reactions to it…I’m happy being judged by what I do instead.”* (Male, 40s)  *“After while I have taught myself to say that I am on disability for mental health reasons, done deal(…)Have had good experiences with that.”* (Male, 40s)  *“I have been very concerned with not wanting to use the experience I have of being sick as a conversational ice breaker, that it’s supposed to start a conversation(…)I don’t want to pull the mental illness-card.”* (Female, 30s) |
| Stigma and schizophrenia | *“I think it’s really hard to talk about the diagnosis too, because I think there is a lot of prejudice and you get worried when you hear about it(...) On the* *other* *hand there’s a lot more openness about it now, so when I hear someone talk about having psychosis on TV …I really recognize it.”* (Male, 40s)  *“Some are brave and come out publicly, but not many with schizophrenia, although some have come out with bipolar, but the time might not be ripe yet.”* (Female, 50s) |

1. Haavind H. Liten og stor: mødres omsorg og barns utviklingsmuligheter [The big and the little one: Maternal care and the developmental possibilities for children]. Oslo: Universitetsforlaget; 1987.

2. Jansen A. Positioning and subjectivation in research interviews: why bother talking to a researcher? Int J Soc Res Methodol [Internet]. 2015;18(1):27–39. Available from: http://dx.doi.org/10.1080/13645579.2013.845711
